# Synthesis, crystal structure, Hirshfeld surface analysis and DFT study of the 1,1′-(buta-1,3-diyne-1,4-di­yl)bis­(cyclo­hexan-1-ol)

**DOI:** 10.1107/S2056989023004772

**Published:** 2023-06-02

**Authors:** Sarvinoz I. Tirkasheva, Odiljon E. Ziyadullaev, Alisher G. Eshimbetov, Bakhtiyar T. Ibragimov, Jamshid M. Ashurov

**Affiliations:** a Chirchik State Pedagogical University, 111700, A. Temur Str. 104, Chirchik, Uzbekistan; b Institute of Bioorganic Chemistry, Academy of Sciences of Uzbekistan, 100125, M. Ulugbek Str 83, Tashkent, Uzbekistan; University of Durham, United Kingdom

**Keywords:** synthesis, 1,1′-(buta-1,3-diyne-1,4-di­yl)bis­(cyclo­hexan-1-ol), crystal structure, hydrogen bond, Hirshfeld surface analysis, DFT study.

## Abstract

The title compound, C_16_H_22_O_2_, was synthesized in order to obtain its guest-free form because ‘wheel-and-axle’-shaped mol­ecules tend to crystallize from solutions as solvates or host–guest mol­ecules. It crystallizes in the monoclinic space group *P*2/*c* with two crystallographically non-equivalent mol­ecules, one situated on an inversion center and the other on a twofold axis. The rod-like 1,3-diyne fragments have the usual linear geometry. In the crystal, O—H ⋯ O bonds form eight-membered rings of the *R* ^4^_4_(8) type, linking mol­ecules into layers. The Hirshfeld surface analysis indicates that the largest con­tributions are from inter­molecular H⋯H (*ca* 71%) and H⋯C/C⋯H (*ca* 19%) contacts. The energies of the frontier mol­ecular orbitals were determined by DFT calculations at the B3LYP/def2-TZVP level of theory.

## Abstract 2

The two crystallographically non-equivalent mol­ecules in the title compound have *C*_2_ and *C_i_* symmetries. The crystal structure features strong inter­mol­ecular O—H⋯O hydrogen bonds, which form eight-membered rings with 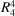 (8) graph-set motifs, linking the mol­ecules into layers.

## 1. Chemical context

The presence of two triple C≡C bonds and two hy­droxy groups in the mol­ecules of di­acetyl­ene diols *R* ^1^ *R* ^2^(OH)C—C≡C—C≡C—C(OH)*R* ^3^ *R* ^4^, as well as substituents with different structures and functional groups containing heteroatoms, increases the possibilities of synthesis and the production of valuable, chemically stable and biologically active compounds based on such compounds (Cadierno, 2022 ▸). In particular, as the hydrogen atom adjacent to the strong C≡C bond is labile (Brücner, 2010 ▸), terminal alkynes easily undergo nucleophilic addition reactions to the carbonyl group and terminal (Hosseini *et al.*, 2020 ▸; Sum *et al.*, 2013 ▸) or inter­nal acetyl­ene alcohols (Tanaka *et al.*, 2011 ▸; Motoki *et al.*, 2007 ▸) and diols (Ardila-Fierro *et al.*, 2019 ▸) with various substituents. Di­acetyl­ene diols and polyacetyl­ene diols (Shi Shun *et al.*, 2006 ▸) can also be synthesized by performing dimerization processes. Many reactions, such as cyclization (Zhang *et al.*, 2010 ▸) or substitution (Kuang *et al.*, 2018 ▸), based on the hy­droxy group (–OH) or its hydrogen atom in an acetyl­ene alcohol, give opportunities to synthesize new biologically active substances. Hexa-2,4-diene-1,6-diol and its derivatives have been found to have anti­cancer chemotherapeutic properties (Lee *et al.*, 2015 ▸). Moreover, some di­acetyl­ene diols and their derivatives have anti­bacterial (Ankisetty *et al.*, 2012 ▸), anti­viral (Geng *et al.*, 2015 ▸) and neuritogenic (Wang *et al.*, 2011 ▸) activities.

**Figure 1:**
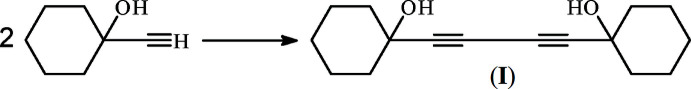
Synthesis of compound (I).

Moreover, the above indicated substances behave as versatile host compounds accommodating many guest species (Weber *et al.*, 1991 ▸) because the shape of their mol­ecules is inefficient for close packing in crystals. Therefore, the preparation of such compounds in their pure form, *i.e.* a guest-free state, is of inter­est. This paper describes the preparation (Fig. 1 ▸), mol­ecular and crystal structure, as well Hirshfeld surface analysis of the guest-free crystal of the title compound, (I). 
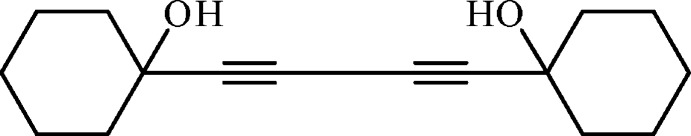


## 2. Structural commentary

**Figure 2:**
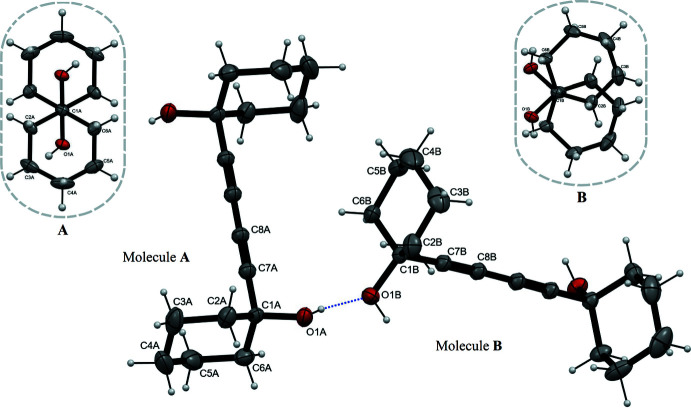
The mol­ecular structure of (I). Displacement ellipsoids are drawn at the 30% probability level, hydrogen bonds are shown as dotted lines. Symmetrically independent atoms are labelled, the rest are generated by the symmetry operations 1 − *x*, 1 − *y*, 1 − *z* (for *A*) and −*x*, *y*, 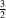 − *z* (for *B*).

There are the principles of directed host design formulated by Weber (Weber *et al.*, 1991 ▸), according to which bulky and rigid compounds are packed in crystals inefficiently, leaving suitable cavities for the accommodation of guest mol­ecules. Indeed, host compounds with a ‘wheel-and-axle’ shape of the mol­ecule easily include several guests (Weber *et al.*, 2004 ▸). However, in the case of compound (I) belonging to this family, only one inclusion compound (with 1,4-di­aza­bicyclo-[2.2.2]octane as the guest) has been structurally characterized (Chandrasekhar *et al.*, 2013 ▸). In our experimental conditions we have obtained guest-free crystals of (I). They belong to the monoclinic system with space group *P*2/*c*. There are two crystallographically non-equivalent mol­ecules, both situated on symmetry elements: mol­ecule *A* is located on an inversion center while mol­ecule *B* lies on a twofold axis. Thus there are two half-mol­ecules in the asymmetric part of the unit cell. The rod-like 1,3-diyne fragment has the usual linear geometry and bond lengths (Weber *et al.*, 1991 ▸, 2004 ▸; Chandrasekhar *et al.*, 2013 ▸). The mol­ecular structure of (I) is shown in Fig. 2 ▸. The cyclo­hexane moieties of both independent mol­ecules adopt chair conformations, with atoms C1 and C4 deviating from the plane of the remaining four atoms by 0.655 and −0.657 Å, respectively, in mol­ecule *A*, by 0.668 and −0.638 Å in *B*. The disposition of the cyclo­hexane rings relative to the 1,3-diyne chain is the same in mol­ecules *A* and *B*, as shown by the similar distances C7*A*⋯*Cg*1 = 2.331 Å and C7*B*⋯*Cg*2 = 2.329 Å where *Cg*1 and *Cg*2 are the ring centroids. However, the orientation of the rings relative to each other is different (Fig. 2 ▸, inserts): *trans* in mol­ecule *A*, *gauche* in *B*, both different from the nearly eclipsed disposition in the one known mol­ecular complex of (I).

## 3. Supra­molecular features

**Figure 3:**
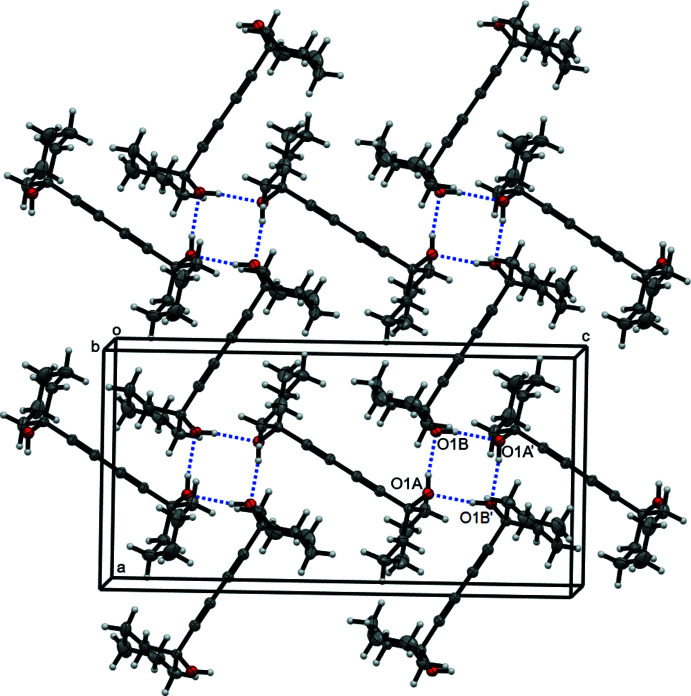
Packing diagram of (I). Dotted lines indicate hydrogen bonds. Symmetry operation for primed atoms: 1 − *x*, *y*, 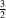 − *z.*

The mol­ecule of (I) has two OH groups. Each group realises its proton-donor and proton-acceptor possibilities, forming inter­molecular hydrogen bonds (Table 1 ▸) O1*A*—H1*A*⋯O1*B* and O1*B*—H1*B*⋯O1*A* with O⋯O distances of 2.748 (1) and 2.771 (1) Å, respectively. As shown in Fig. 3 ▸, each mol­ecule participates in two 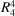 (8) rings of hydrogen bonds (Grell *et al.*, 1999 ▸), each ring involving two mol­ecules of type *A* and two of *B*. These bonds give rise to a two-dimensional supra­molecular layer parallel to the *ac* plane. The layers are incorporated into a three-dimensional network by van der Waals inter­actions (Fig. 3 ▸).

## 4. Hirshfeld surface analysis

**Figure 4:**
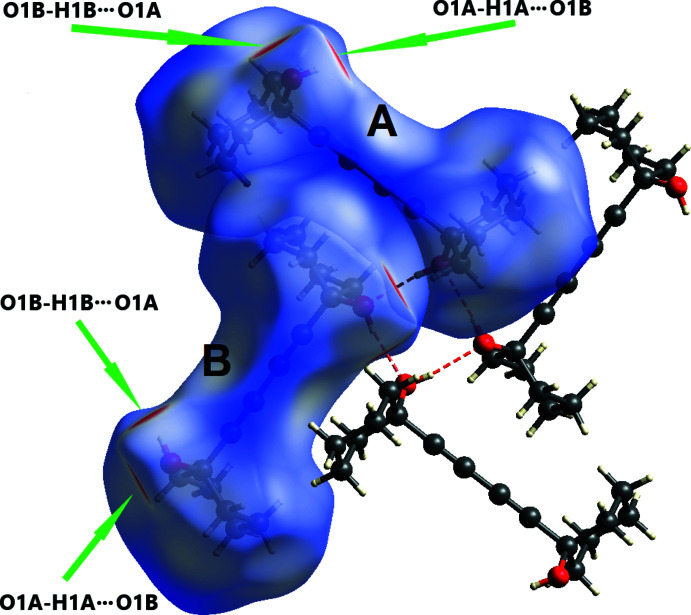
Three-dimensional Hirshfeld surfaces of mol­ecules *A* and *B* of (I) plotted over *d*_norm_ in the range −0.5154 to 1.9215 a.u.

**Figure 5:**
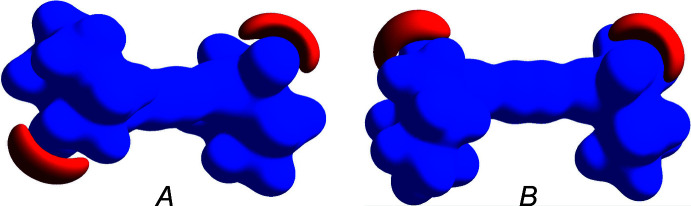
Hirshfeld surfaces of mol­ecules *A* and *B* plotted over electrostatic potential in the range −0.05 to 0.05 a.u. using the B3LYP/6–311 G(d,p) basis set at the Hartree–Fock level of theory. Blue and red regions indicate positive and negative potentials, respectively.

**Figure 6:**
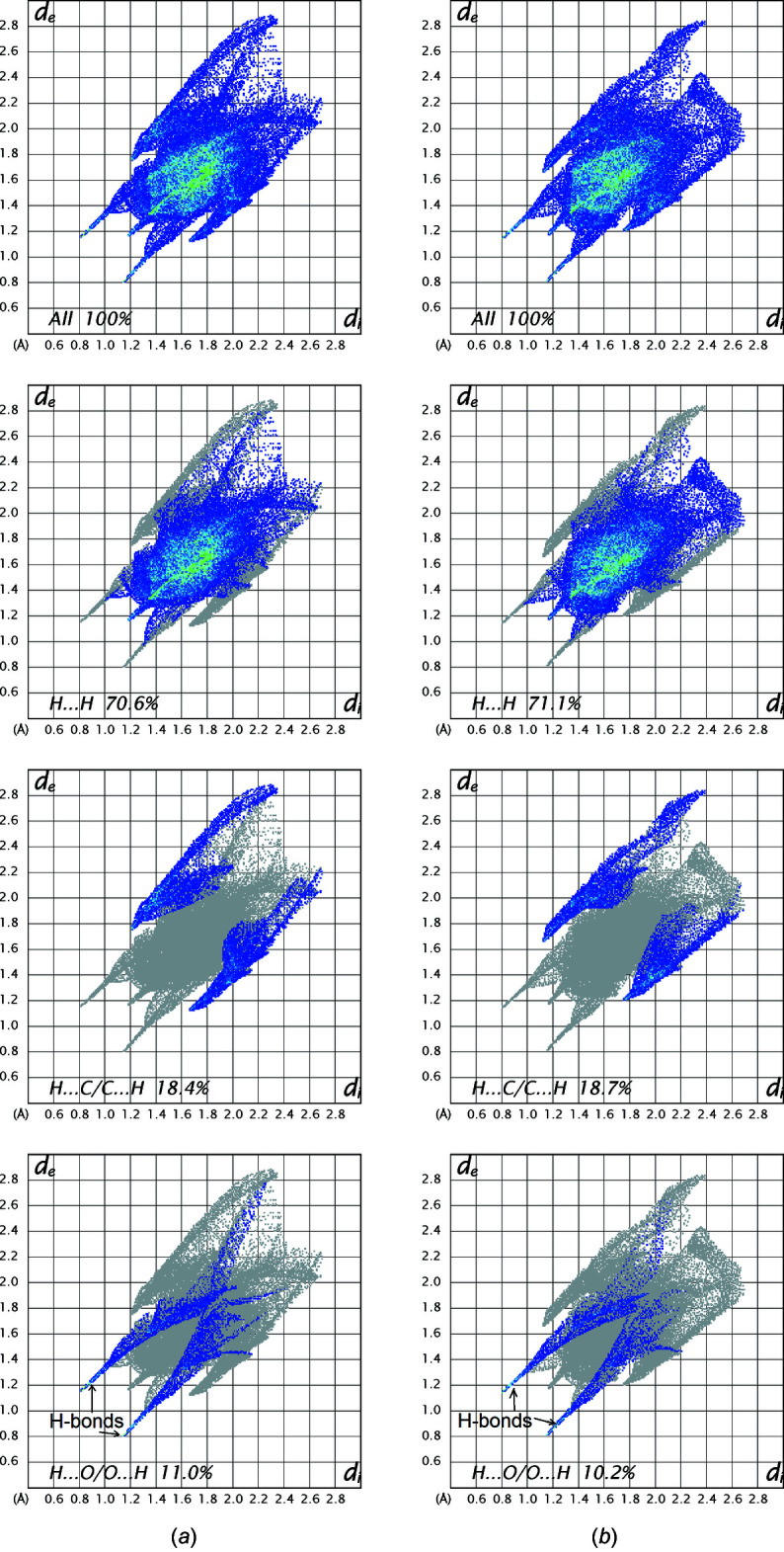
Complete two-dimensional fingerprint plots for mol­ecules *A* (*a*) and *B* (*b*) of (I) with relative contributions of individual contacts. Note the ‘spikes’ indicating strong hydrogen bonds.

Hirshfeld surfaces were calculated and two-dimensional fingerprints generated using *CrystalExplorer21* (Spackman *et al.*, 2021 ▸). Hirshfeld surfaces were obtained using a standard (high) surface resolution with the three-dimensional *d*_norm_ surfaces mapped over a fixed color scale of −0.5154 (red) to 1.9215 (blue) (Fig. 4 ▸). The only red spots on the surface (revealing strong inter­actions) correspond to the O—H⋯O hydrogen bonds, the rest representing standard (white) or longer than standard (blue) van der Waals contacts. This agrees with the calculated electrostatic potential of the mol­ecule (Fig. 5 ▸) where the only negative potential (acceptor) areas are around the O atoms. The two-dimensional fingerprint plots (in *d*_e_ *vs d*_i_ coordinates) (Fig. 6 ▸) show that mol­ecules *A* and *B* have very similar environments, the major contributions being from contacts H⋯H (70.6 for *A*, 71.1% for *B*), H⋯C/C⋯H (18.4 and 18.7%) and H⋯O/O⋯H (11.0 and 10.2%).

## 5. The analysis of DFT calculations

**Figure 7:**
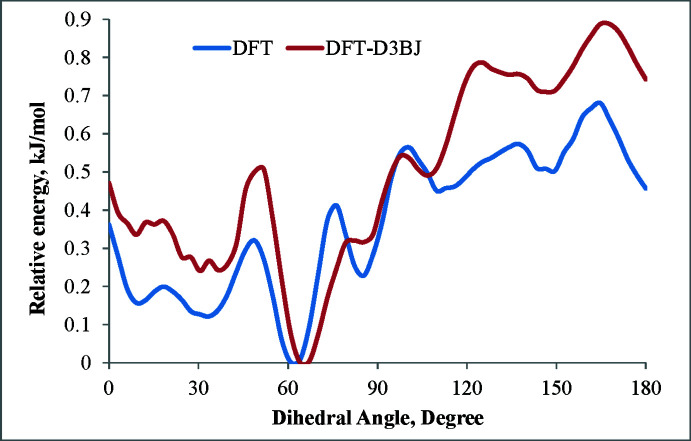
Potential energy curve for mol­ecule (I) as a function of the dihedral angle ω.

**Figure 8:**
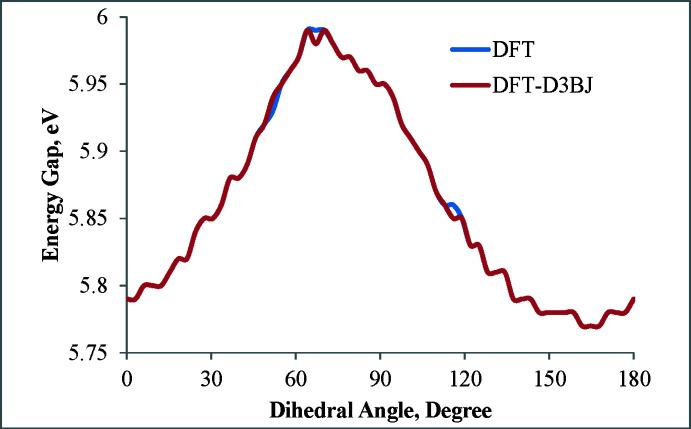
The HOMO–LUMO energy gap of mol­ecule (I) as a function of ω.

**Figure 9:**
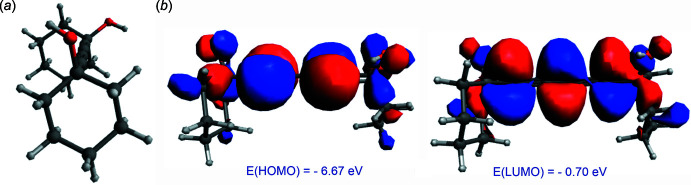
(*a*) Optimized conformation (ω = 61°) of (I) and (*b*) electron densities on its frontier MOs by the DFT/def2-TZVP method.

The co-presence of *trans* and *gauche* conformations of (I) in the crystal was mentioned above. In order to determine the intra­molecular rotational barrier of a cyclo­hexan-1-ol fragment around the diyne rod (*i.e.* the C*sp* ^3^—C*sp* bond), the relaxed scan calculation has been carried out in a vacuum by B3LYP/def2-TZVP method using the *ORCA* program package (Neese, 2022 ▸). The initial geometry of (I) was taken from the crystal structure (CIF file) and the input files were prepared using *Avogadro* program package (Hanwell *et al.*, 2012 ▸). The O1—C1⋯C1′—O1′ torsion angle (ω) was varied from 0 to 180° in 3° steps with full optimization of the mol­ecular geometry at each step. Then single-point calculations were performed using the B3LYP-D3BJ/def2-TZVP basis set for the geometries obtained at each step, by including dispersion corrections (Grimme *et al.*, 2011 ▸). Thus we observed energy minima at ω = 9, 30, 61, 85, 109, 146 and 180° (Fig. 7 ▸), the deepest one being at 61° by DFT/def2-TZVP calculations (or 64° by DFT-D3BJ/def2-TZVP); however, the rotation barrier was low, 0.7 or 0.9 kJ mol^−1^1, respectively. Thus, an easy transition between conformations can occur in solution and, apparently, the inter­molecular (packing) inter­actions played a decisive role in the implementation of the *gauche* (ω = 85°) and *trans* (ω = 180°) conformations in the crystal. To study the influence of ω variation on the electronic parameters, we analyzed the changes of HOMO and LUMO energies, and the energy gap upon varying ω from 0 to 180°. The energy and electron density at these orbitals are important in defining the mol­ecule’s chemistry (Fukui, 1982 ▸; Hoffmann *et al.*, 1965 ▸), the HOMO correlating with the ionization potential and representing the electron-donating ability of a mol­ecule, while the LUMO correlates with the electron affinity of a mol­ecule and represents its electron-accepting ability. The energy difference (energy gap) between HOMO and LUMO is known to represent the stability or reactivity of a mol­ecule in a series of related compounds (Pearson, 1988 ▸; Jahnke *et al.*, 2010 ▸). For (I), the HOMO and LUMO energies and the energy gap change slightly with ω, the former varying from −6.63 to −6.72 eV and the latter from −0.69 to −0.84 eV, while the energy gap varies from 5.79 to 5.99 eV (Fig. 8 ▸). The widest energy gap (5.99 eV) was found at energetically optimal conformation with ω = 61 or 64° (*vide supra*). Mol­ecule (I) has a low-lying HOMO and a high-lying LUMO and consequently a wide HOMO–LUMO gap, which indicates the high thermodynamic stability and low reactivity of the mol­ecule. Despite this, the highly unsaturated carbon chains could also exhibit various reaction properties (photoisomerization, nucleophilic addition of alcohols, thiols and amines to the triple bond) under special conditions (Shi *et al.*, 2014 ▸). The reactivity of (I) toward nucleophiles can be inferred from the electron density on LUMO, which is predominantly the π* orbital of di­acetyl­ene C atoms (Fig. 9 ▸). The HOMO is a π-type MO and is mainly delocalized along the di­acetyl­ene fragment (Fig. 9 ▸). However, these atoms are unlikely to have an electron-donating ability to electrophile reagents because of the low-lying HOMO.

Thus, theoretical calculations showed that the rotation of hexa­nol-1 fragment around the C*sp* ^3^—C*sp* bond can pass through several conformational minima that differ in ω. However, all these conformations make a negligible difference to the total energies and the rotational barrier between them. The conformations observed in the crystal packing arose as a result of the action of inter­molecular inter­action forces.

## 6. Database survey

A survey of the Cambridge Structural Database (*CONQUEST* version 2021 3.0; Groom *et al.*, 2016 ▸) revealed 198 structures in which an OH group and any other substituents are attached to each end of a hexa-2,4-diyne rod. However, the only compound involving compound (I) is its complex with 1,4-di­aza­bicyclo [2.2.2]octane (MIRJEE; Chandrasekhar *et al.*, 2013 ▸). The existence of this co-crystal could be expected from the propensity of ‘wheel-and-axle’-shaped mol­ecules to form host–guest structures.

## 7. Synthesis and crystallization

The dimerization process of 1-ethynyl­cyclo­hexa­nol was conducted at 298 K for 48 h, based on a catalytic system with a copper(I) chloride catalyst, tetra­chloro­methane, *N* ^1^,*N* ^1^,*N* ^2^,*N* ^2^-tetra­methyl­ethylenedi­amine as a ligand and ethanol as the solvent, following the general routine used by Tirkasheva *et al.* (2022 ▸) to prepare 8,13-di­methyl­cosa-9,11-diyne-8,13-diol. This yielded 1,1′-(buta-1,3-diyne-1,4-di­yl)bis­(cyclo­hexan-1-ol) (I) as a brown liquid. 25 mg (0.1 mmol) of (I) were dissolved in 2 ml of chloro­form in a 50 ml round-bottom flask and the solvent was removed under vacuum. After the chloro­form was completely removed, 2 ml of CH_2_Cl_2_ and 1 ml of methanol were added to the flask. Brown single crystals of the title compound suitable for X-ray diffraction analysis were grown over three days by slow evaporation of the solvent, yield 76%, m.p. 448 K. Elemental analysis for C_16_H_22_O_2_ (246.33): calculated C 78.01; H 9.00%; found C 77.95; H 8.94%. FTIR (ATR), cm^−1^: 3326 (OH).

## 8. Refinement

Crystal data, data collection and structure refinement details are summarized in Table 2 ▸. All H atoms were positioned geometrically, C—H 0.97 Å (methyl­ene), O—H 0.82 Å (hydroxyl group) and refined as riding with *U*_iso_(H) = 1.2*U*_eq_(C) or 1.5*U*_eq_(O).

## Supplementary Material

Crystal structure: contains datablock(s) I. DOI: 10.1107/S2056989023004772/zv2024sup1.cif

Structure factors: contains datablock(s) I. DOI: 10.1107/S2056989023004772/zv2024Isup2.hkl

Supporting information file. DOI: 10.1107/S2056989023004772/zv2024Isup3.cml

CCDC reference: 2189423

Additional supporting information:  crystallographic information; 3D view; checkCIF report

## Figures and Tables

**Table 1:** Hydrogen-bond geometry (Å, °)

*D*—H⋯*A*	*D*—H	H⋯*A*	*D*⋯*A*	*D*—H⋯*A*
O1*B*—H1*B*⋯O1*A* ^i^	0.82	1.96	2.7709 (15)	168
O1*A*—H1*A*⋯O1*B*	0.82	1.94	2.7481 (15)	168

Symmetry code: (i) 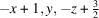 .

**Table 2:** Experimental details

Crystal data	
Chemical formula	C_16_H_22_O_2_
*M*_r_	246.33
Crystal system, space group	Monoclinic, *P*2/*c*
Temperature (K)	293
*a*, *b*, *c* (Å)	10.4134 (2), 6.9167 (2), 20.4801 (5)
β (°)	90.308 (2)
*V* (Å^3^)	1475.09 (6)
*Z*	4
Radiation type	Cu *K*α
μ (mm^−1^)	0.56
Crystal size (mm)	0.30 × 0.24 × 0.15

Data collection	
Diffractometer	XtaLAB Synergy, Single source at home/near, HyPix3000
Absorption correction	Multi-scan (*CrysAlis PRO*; Rigaku OD, 2020 ▸)
*T*_min_, *T*_max_	0.960, 1.000
No. of measured, independent and observed [*I* > 2σ(*I*)] reflections	13939, 2866, 2161
*R*_int_	0.038
(sin θ/λ)_max_ (Å^−1^)	0.615

Refinement	
*R*[*F* ^2^ > 2σ(*F* ^2^)], *wR*(*F* ^2^), *S*	0.044, 0.129, 1.07
No. of reflections	2866
No. of parameters	166
H-atom treatment	H-atom parameters constrained
Δρ_max_, Δρ_min_ (e Å^−3^)	0.12, −0.16

Computer programs: *CrysAlis PRO* (Rigaku OD, 2020 ▸), *SHELXT2018/2* (Sheldrick, 2015*a* ▸), *SHELXL2019/2* (Sheldrick, 2015*b* ▸), *Mercury* (Macrae *et al.*, 2020 ▸) and *publCIF* (Westrip, 2010 ▸).

## supplementary crystallographic information

### Crystal data

**Table:**

C_16_H_22_O_2_	*F*(000) = 536
*M**_r_* = 246.33	*D*_x_ = 1.109 Mg m^−^^3^
Monoclinic, *P*2/*c*	Cu *K*α radiation, λ = 1.54184 Å
*a* = 10.4134 (2) Å	Cell parameters from 4018 reflections
*b* = 6.9167 (2) Å	θ = 4.2–70.2°
*c* = 20.4801 (5) Å	µ = 0.56 mm^−^^1^
β = 90.308 (2)°	*T* = 293 K
*V* = 1475.09 (6) Å^3^	Block, colorless
*Z* = 4	0.30 × 0.24 × 0.15 mm

### Data collection

**Table:**

XtaLAB Synergy, Single source at home/near, HyPix3000 diffractometer	2866 independent reflections
Radiation source: micro-focus sealed X-ray tube, PhotonJet (Cu) X-ray Source	2161 reflections with *I* > 2σ(*I*)
Mirror monochromator	*R*_int_ = 0.038
Detector resolution: 10.0000 pixels mm^-1^	θ_max_ = 71.5°, θ_min_ = 4.3°
ω scans	*h* = −12→12
Absorption correction: multi-scan (CrysAlisPro; Rigaku OD, 2020)	*k* = −8→8
*T*_min_ = 0.960, *T*_max_ = 1.000	*l* = −25→25
13939 measured reflections	

### Refinement

**Table:**

Refinement on *F*^2^	Hydrogen site location: inferred from neighbouring sites
Least-squares matrix: full	H-atom parameters constrained
*R*[*F*^2^ > 2σ(*F*^2^)] = 0.044	*w* = 1/[σ^2^(*F*_o_^2^) + (0.0565*P*)^2^ + 0.2398*P*] where *P* = (*F*_o_^2^ + 2*F*_c_^2^)/3
*wR*(*F*^2^) = 0.129	(Δ/σ)_max_ = 0.001
*S* = 1.07	Δρ_max_ = 0.12 e Å^−^^3^
2866 reflections	Δρ_min_ = −0.16 e Å^−^^3^
166 parameters	Extinction correction: *SHELXL2019/2* (Sheldrick, 2015b), Fc^*^=kFc[1+0.001xFc^2^λ^3^/sin(2θ)]^-1/4^
0 restraints	Extinction coefficient: 0.0018 (4)

### Special details

**Table:**

Geometry. All esds (except the esd in the dihedral angle between two l.s. planes) are estimated using the full covariance matrix. The cell esds are taken into account individually in the estimation of esds in distances, angles and torsion angles; correlations between esds in cell parameters are only used when they are defined by crystal symmetry. An approximate (isotropic) treatment of cell esds is used for estimating esds involving l.s. planes.

### Fractional atomic coordinates and isotropic or equivalent isotropic displacement parameters (Å^2^)

**Table:**

	*x*	*y*	*z*	*U*_iso_*/*U*_eq_	
O1A	0.61299 (10)	0.28504 (18)	0.67530 (5)	0.0500 (3)	
H1A	0.534651	0.297081	0.675485	0.075*	
O1B	0.35098 (10)	0.28922 (18)	0.69056 (5)	0.0514 (3)	
H1B	0.352776	0.275754	0.730331	0.077*	
C8B	0.05502 (14)	0.1260 (2)	0.73133 (7)	0.0436 (4)	
C7B	0.15086 (14)	0.1312 (2)	0.69953 (7)	0.0428 (4)	
C7A	0.59587 (14)	0.4643 (3)	0.57736 (7)	0.0438 (4)	
C1A	0.66899 (13)	0.4401 (2)	0.63846 (7)	0.0396 (4)	
C1B	0.27132 (13)	0.1416 (2)	0.66200 (7)	0.0393 (4)	
C8A	0.53529 (14)	0.4875 (2)	0.52842 (7)	0.0454 (4)	
C6B	0.24442 (16)	0.2025 (3)	0.59194 (7)	0.0515 (4)	
H6BA	0.325069	0.226105	0.569837	0.062*	
H6BB	0.195840	0.322206	0.591871	0.062*	
C2A	0.80687 (15)	0.3812 (3)	0.62439 (9)	0.0572 (5)	
H2AA	0.806869	0.268548	0.596191	0.069*	
H2AB	0.849339	0.346281	0.664971	0.069*	
C6A	0.66516 (19)	0.6273 (3)	0.67772 (8)	0.0578 (5)	
H6AA	0.576503	0.666216	0.683656	0.069*	
H6AB	0.702445	0.604832	0.720557	0.069*	
C2B	0.33965 (16)	−0.0527 (3)	0.66322 (10)	0.0581 (5)	
H2BA	0.423514	−0.038974	0.643367	0.070*	
H2BB	0.352351	−0.092677	0.708192	0.070*	
C5B	0.1692 (2)	0.0480 (4)	0.55515 (9)	0.0738 (6)	
H5BA	0.084237	0.036283	0.573930	0.089*	
H5BB	0.159152	0.086946	0.509906	0.089*	
C3B	0.2648 (2)	−0.2066 (3)	0.62734 (12)	0.0791 (7)	
H3BA	0.184876	−0.230967	0.650026	0.095*	
H3BB	0.313885	−0.325736	0.627015	0.095*	
C3A	0.88123 (18)	0.5442 (4)	0.59165 (11)	0.0813 (7)	
H3AA	0.845971	0.567268	0.548409	0.098*	
H3AB	0.970324	0.506143	0.586823	0.098*	
C5A	0.7378 (2)	0.7890 (3)	0.64418 (11)	0.0819 (7)	
H5AA	0.695858	0.820426	0.603132	0.098*	
H5AB	0.736769	0.903581	0.671491	0.098*	
C4A	0.8742 (3)	0.7302 (4)	0.63159 (13)	0.0994 (9)	
H4AA	0.918322	0.711258	0.672939	0.119*	
H4AB	0.917728	0.833008	0.608294	0.119*	
C4B	0.2359 (3)	−0.1460 (4)	0.55798 (13)	0.0955 (9)	
H4BA	0.181703	−0.242772	0.537411	0.115*	
H4BB	0.315449	−0.139078	0.533687	0.115*	

### Atomic displacement parameters (Å^2^)

**Table:**

	*U*^11^	*U*^22^	*U*^33^	*U*^12^	*U*^13^	*U*^23^
O1A	0.0444 (6)	0.0596 (7)	0.0460 (6)	−0.0077 (5)	−0.0005 (5)	0.0128 (5)
O1B	0.0458 (6)	0.0646 (8)	0.0439 (6)	−0.0126 (5)	0.0007 (5)	−0.0067 (6)
C8B	0.0370 (7)	0.0557 (10)	0.0381 (8)	−0.0001 (7)	0.0016 (6)	−0.0007 (7)
C7B	0.0391 (8)	0.0511 (9)	0.0382 (8)	−0.0013 (7)	0.0020 (6)	−0.0006 (7)
C7A	0.0400 (8)	0.0532 (10)	0.0381 (8)	−0.0031 (7)	−0.0021 (6)	0.0029 (7)
C1A	0.0371 (7)	0.0488 (9)	0.0327 (7)	−0.0037 (6)	−0.0029 (6)	0.0055 (6)
C1B	0.0336 (7)	0.0472 (9)	0.0372 (8)	−0.0037 (6)	0.0038 (6)	−0.0016 (6)
C8A	0.0410 (8)	0.0567 (10)	0.0382 (8)	−0.0043 (7)	−0.0039 (6)	0.0053 (7)
C6B	0.0540 (9)	0.0611 (11)	0.0394 (8)	−0.0133 (8)	0.0027 (7)	0.0063 (8)
C2A	0.0415 (8)	0.0768 (13)	0.0533 (10)	0.0059 (8)	0.0012 (7)	0.0159 (9)
C6A	0.0735 (12)	0.0561 (11)	0.0438 (9)	−0.0029 (9)	−0.0048 (8)	−0.0029 (8)
C2B	0.0479 (9)	0.0556 (11)	0.0708 (12)	0.0078 (8)	0.0113 (8)	0.0058 (9)
C5B	0.0848 (14)	0.0970 (17)	0.0396 (9)	−0.0355 (13)	−0.0045 (9)	0.0000 (10)
C3B	0.0827 (14)	0.0484 (11)	0.1065 (19)	−0.0005 (10)	0.0212 (13)	−0.0140 (12)
C3A	0.0423 (10)	0.120 (2)	0.0817 (14)	−0.0086 (11)	0.0073 (9)	0.0352 (15)
C5A	0.121 (2)	0.0562 (13)	0.0686 (13)	−0.0264 (13)	−0.0130 (13)	−0.0001 (10)
C4A	0.0975 (19)	0.114 (2)	0.0869 (17)	−0.0640 (17)	−0.0204 (14)	0.0256 (16)
C4B	0.112 (2)	0.0905 (19)	0.0837 (17)	−0.0338 (16)	0.0247 (14)	−0.0436 (15)

### Geometric parameters (Å, º)

**Table:**

O1A—H1A	0.8200	C6A—H6AB	0.9700
O1A—C1A	1.4365 (18)	C6A—C5A	1.517 (3)
O1B—H1B	0.8200	C2B—H2BA	0.9700
O1B—C1B	1.4379 (18)	C2B—H2BB	0.9700
C8B—C8B^i^	1.381 (3)	C2B—C3B	1.508 (3)
C8B—C7B	1.195 (2)	C5B—H5BA	0.9700
C7B—C1B	1.4764 (19)	C5B—H5BB	0.9700
C7A—C1A	1.4709 (19)	C5B—C4B	1.512 (4)
C7A—C8A	1.192 (2)	C3B—H3BA	0.9700
C1A—C2A	1.521 (2)	C3B—H3BB	0.9700
C1A—C6A	1.525 (2)	C3B—C4B	1.510 (4)
C1B—C6B	1.520 (2)	C3A—H3AA	0.9700
C1B—C2B	1.521 (2)	C3A—H3AB	0.9700
C8A—C8A^ii^	1.384 (3)	C3A—C4A	1.526 (4)
C6B—H6BA	0.9700	C5A—H5AA	0.9700
C6B—H6BB	0.9700	C5A—H5AB	0.9700
C6B—C5B	1.522 (3)	C5A—C4A	1.501 (4)
C2A—H2AA	0.9700	C4A—H4AA	0.9700
C2A—H2AB	0.9700	C4A—H4AB	0.9700
C2A—C3A	1.525 (3)	C4B—H4BA	0.9700
C6A—H6AA	0.9700	C4B—H4BB	0.9700
			
C1A—O1A—H1A	109.5	C3B—C2B—C1B	112.03 (15)
C1B—O1B—H1B	109.5	C3B—C2B—H2BA	109.2
C7B—C8B—C8B^i^	178.17 (13)	C3B—C2B—H2BB	109.2
C8B—C7B—C1B	178.07 (17)	C6B—C5B—H5BA	109.3
C8A—C7A—C1A	178.52 (18)	C6B—C5B—H5BB	109.3
O1A—C1A—C7A	108.79 (12)	H5BA—C5B—H5BB	107.9
O1A—C1A—C2A	106.62 (13)	C4B—C5B—C6B	111.67 (18)
O1A—C1A—C6A	110.19 (12)	C4B—C5B—H5BA	109.3
C7A—C1A—C2A	110.74 (13)	C4B—C5B—H5BB	109.3
C7A—C1A—C6A	109.69 (14)	C2B—C3B—H3BA	109.4
C2A—C1A—C6A	110.76 (14)	C2B—C3B—H3BB	109.4
O1B—C1B—C7B	108.22 (12)	C2B—C3B—C4B	111.26 (19)
O1B—C1B—C6B	106.85 (12)	H3BA—C3B—H3BB	108.0
O1B—C1B—C2B	110.61 (13)	C4B—C3B—H3BA	109.4
C7B—C1B—C6B	110.61 (12)	C4B—C3B—H3BB	109.4
C7B—C1B—C2B	110.33 (13)	C2A—C3A—H3AA	109.4
C6B—C1B—C2B	110.15 (14)	C2A—C3A—H3AB	109.4
C7A—C8A—C8A^ii^	179.4 (2)	C2A—C3A—C4A	111.19 (18)
C1B—C6B—H6BA	109.4	H3AA—C3A—H3AB	108.0
C1B—C6B—H6BB	109.4	C4A—C3A—H3AA	109.4
C1B—C6B—C5B	111.36 (15)	C4A—C3A—H3AB	109.4
H6BA—C6B—H6BB	108.0	C6A—C5A—H5AA	109.5
C5B—C6B—H6BA	109.4	C6A—C5A—H5AB	109.5
C5B—C6B—H6BB	109.4	H5AA—C5A—H5AB	108.1
C1A—C2A—H2AA	109.3	C4A—C5A—C6A	110.6 (2)
C1A—C2A—H2AB	109.3	C4A—C5A—H5AA	109.5
C1A—C2A—C3A	111.56 (16)	C4A—C5A—H5AB	109.5
H2AA—C2A—H2AB	108.0	C3A—C4A—H4AA	109.3
C3A—C2A—H2AA	109.3	C3A—C4A—H4AB	109.3
C3A—C2A—H2AB	109.3	C5A—C4A—C3A	111.62 (18)
C1A—C6A—H6AA	109.2	C5A—C4A—H4AA	109.3
C1A—C6A—H6AB	109.2	C5A—C4A—H4AB	109.3
H6AA—C6A—H6AB	107.9	H4AA—C4A—H4AB	108.0
C5A—C6A—C1A	111.90 (15)	C5B—C4B—H4BA	109.3
C5A—C6A—H6AA	109.2	C5B—C4B—H4BB	109.3
C5A—C6A—H6AB	109.2	C3B—C4B—C5B	111.81 (18)
C1B—C2B—H2BA	109.2	C3B—C4B—H4BA	109.3
C1B—C2B—H2BB	109.2	C3B—C4B—H4BB	109.3
H2BA—C2B—H2BB	107.9	H4BA—C4B—H4BB	107.9
			
O1A—C1A—C2A—C3A	−173.86 (15)	C1B—C6B—C5B—C4B	−54.8 (2)
O1A—C1A—C6A—C5A	173.01 (15)	C1B—C2B—C3B—C4B	55.6 (2)
O1B—C1B—C6B—C5B	175.44 (15)	C6B—C1B—C2B—C3B	−56.09 (19)
O1B—C1B—C2B—C3B	−173.98 (14)	C6B—C5B—C4B—C3B	54.0 (2)
C7B—C1B—C6B—C5B	−67.0 (2)	C2A—C1A—C6A—C5A	55.3 (2)
C7B—C1B—C2B—C3B	66.31 (19)	C2A—C3A—C4A—C5A	−55.5 (3)
C7A—C1A—C2A—C3A	67.9 (2)	C6A—C1A—C2A—C3A	−54.0 (2)
C7A—C1A—C6A—C5A	−67.3 (2)	C6A—C5A—C4A—C3A	56.3 (3)
C1A—C2A—C3A—C4A	54.2 (2)	C2B—C1B—C6B—C5B	55.26 (19)
C1A—C6A—C5A—C4A	−56.5 (2)	C2B—C3B—C4B—C5B	−54.2 (3)

Symmetry codes: (i) −*x*, *y*, −*z*+3/2; (ii) −*x*+1, −*y*+1, −*z*+1.

### Hydrogen-bond geometry (Å, º)

**Table:**

*D*—H···*A*	*D*—H	H···*A*	*D*···*A*	*D*—H···*A*
O1*B*—H1*B*···O1*A*^iii^	0.82	1.96	2.7709 (15)	168
O1*A*—H1*A*···O1*B*	0.82	1.94	2.7481 (15)	168

Symmetry code: (iii) −*x*+1, *y*, −*z*+3/2.
